# Influence of full-length dystrophin on brain volumes in mouse models of Duchenne muscular dystrophy

**DOI:** 10.1371/journal.pone.0194636

**Published:** 2018-03-30

**Authors:** Bauke Kogelman, Artem Khmelinskii, Ingrid Verhaart, Laura van Vliet, Diewertje I. Bink, Annemieke Aartsma-Rus, Maaike van Putten, Louise van der Weerd

**Affiliations:** 1 Department of Radiology, Leiden University Medical Center, Leiden, The Netherlands; 2 Division of Image Processing, Department of Radiology, Leiden University Medical Center, Leiden, the Netherlands; 3 Percuros B.V., Enschede, the Netherlands; 4 Department of Human Genetics, Leiden University Medical Center, Leiden, The Netherlands; 5 Department of Pathology, Academic Medical Center, University of Amsterdam, Amsterdam, The Netherlands; University of Minnesota, UNITED STATES

## Abstract

Duchenne muscular dystrophy (DMD) affects besides muscle also the brain, resulting in memory and behavioral problems. The consequences of dystrophinopathy on gross macroscopic alterations are unclear. To elucidate the effect of full-length dystrophin expression on brain morphology, we used high-resolution post-mortem MRI in mouse models that either express 0% (*mdx*), 100% (BL10) or a low amount of full-length dystrophin (*mdx*-*Xist*^Δhs^). While absence or low amounts of full-length dystrophin did not significantly affect whole brain volume and skull morphology, we found differences in volume of individual brain structures. The results are in line with observations in humans, where whole brain volume was found to be reduced only in patients lacking both full-length dystrophin and the shorter isoform Dp140.

## Introduction

Duchenne muscular dystrophy (DMD) is an X-linked neuromuscular disease characterized by severe and progressive muscle weakness. DMD is caused by frameshift or non-sense mutations in the *DMD* gene preventing synthesis of functional dystrophin proteins. Dystrophin links the intracellular cytoskeleton of muscle fibers to the extracellular matrix, thereby providing structural stability during contractions [1]. In the absence of dystrophin, fibers are more injury-susceptible and replaced by connective and adipose tissue upon exhaustion of the regenerative capacity of the muscle. Consequently, Duchenne patients develop muscle weakness, leading to wheelchair dependency before or in their early teens [2–4]. Respiratory and cardiac function are also affected and despite respiratory support, patients die between the 2^nd^ to 4^th^ decade of life due to cardiorespiratory failure [5].

DMD patients also present with cognitive impairments. They have an intelligence quotient approximately one standard deviation below the mean [6], their verbal short term memory is affected [7], they exhibit long-term memory problems and language impairments [8,9]. Additionally, DMD patients are more prone to epilepsy [10,11] and have a higher incidence for attention deficit hyperactivity disorder (ADHD), autism spectrum disorder and obsessive-compulsive disorder [12–14].

The *DMD* gene has multiple promoters coding for distinct dystrophin isoforms. The full-length isoforms are expressed mainly in muscle (Dp427m) and cortical neurons (Dp427c), whereas the Dp427p isoform was recently found to be only expressed in mouse but not human brain [15]. Besides full-length dystrophin, there are five known smaller isoforms (Dp260, Dp140, Dp116, Dp71 and Dp40) which are all named according to their molecular weight [16–18]. Dp260 is predominately expressed in the retina [19,20], Dp140 is expressed in the central nervous system, retina and kidney, Dp116 is expressed in Schwann cells of the spinal cord and Dp71 and Dp40 are expressed in brain tissue [10,16,21–26]. Cognitive impairment is most prominent in patients with mutations in distal parts of the *DMD* gene, associated with the loss of both full-length and one or multiple of the shorter isoforms [8,27,28].

However, it is unclear what the consequences of dystrophinopathy are on brain morphology [16]. In humans, one magnetic resonance imaging (MRI) study showed a subtle, but significant decrease in whole brain volume in the order of 5% for DMD patients compared to healthy controls [29]. This difference was mainly observed in patients lacking Dp140 in addition to Dp427, consistent with the more severe cognitive phenotype in these patients. Next to this, dystrophinopathy is associated with aberrant skull shape, which was found to be more circular shaped [30].

Studies of brain function and morphology in mouse models of DMD are equally sparse. The *mdx* mouse is the most commonly studied DMD model, which lacks full-length dystrophin due to a point mutation in exon 23 of the murine *Dmd* gene. Similar to patients, absence of Dp427 in the *mdx* mouse is associated with behavioral and social impairments, such as enhanced freezing response in reaction to danger and altered ultrasonic vocal communication [31–33]. Learning and memory performance were also found to be impaired in some studies [34–37], though others report that spatial memory is largely unaffected [38,39]. Brain morphology of *mdx* mice has been reported in two studies [40,41]. Both studies found no significant difference in brain volume between *mdx* and wild type mice. However, these studies were underpowered and the scan resolutions were inadequate to detect the subtle differences in brain volumes found in DMD patients.

The aim of the present study was therefore to investigate the brain morphology of two different DMD mouse models, namely the well-known *mdx* mouse and the *mdx-Xist*^Δhs^ model, which expresses varying low amounts of full-length dystrophin in muscle and brain based on non-random X-inactivation [42,43]. We used high resolution post-mortem MRI followed by automatic segmentation to determine the volume of a large number of brain structures and to assess the skull shape.

## Materials and methods

### Animals

Mice were bred at the animal facility of the Leiden University Medical Center, where they were housed in individually ventilated cages at 20.5°C with 12-hour dark-light cycles and given standard RM3 chow (SDS, Essex, UK) and water *ad libitum*. Post-mortem magnetic resonance imaging (MRI) scans were obtained in n = 10 C57BL/10ScSn-mdx/J (*mdx*), n = 7 C57BL/10ScSnJ (BL10), n = 9 *mdx-Xist*^Δhs^ and n = 9 C57BL/10ScSnJ*-Xist*^Δhs^ (BL10*-Xist*^Δhs^) mice aged three months.

To generate the *mdx-Xist*^Δhs^ model [42], breeding pairs of *mdx* males (carrying a mutated *Dmd* gene) and *Xist*^Δhs^ females (carrying a mutated *Xist* gene) were used. Due to preferential inactivation of the X-chromosome carrying the mutated *Xist* gene, but intact *Dmd* gene, female *mdx-Xist*^Δhs^ offspring express low and varying amounts of full-length dystrophin in skeletal muscle, heart and the brain. *Xist*^Δhs^ mice were also crossed with C57BL/10ScSnJ mice resulting in C57BL/10ScSnJ-*Xist*^Δhs^ wild types to allow comparisons with *mdx-Xist*^Δhs^ mice without genetic background interference. All mice studied were female, inherent to the *mdx-Xist*^Δhs^ strain. To confirm that *mdx*-*Xist*^Δhs^ mice express low levels of full-length dystrophin in the brain, whole brains were isolated of n = 7 *mdx*-*Xist*^Δhs^, n = 1 *Xist*^Δhs^ and n = 1 *mdx* 18-months old female mice for Western blot analyses. Expression levels of Dp427c, Dp427p and Dp71 were assessed in the brains of a different set of 18-months old animals (n = 5 *mdx*, n = 5 BL10 and n = 7 *mdx*-Xist^Δhs^ females). All experiments were approved and executed following EU-guidelines and efforts were made to minimize the burden and distress. Animal welfare was monitored on a daily basis and none of the mice reached the humane endpoints. The brains were harvested after completion of independent studies on the muscles, which were approved by the Animal Experimental Committee of the LUMC (permits 11145 and 12208) and included the approval for sacrifice of the mice.

### Tissue preparation

At the age of three months, mice were anesthetized with either 2% isoflurane or intraperitoneal injection of 200 μL of Hypnorm^®^/Dormicum^®^, depending on availability, and perfused for one minute with 1x phosphate buffer saline (PBS) and for four minutes with 4% paraformaldehyde (PFA). The skull was freed from skin and fat tissue, stored in 4% PFA overnight and was then transferred to 4% PFA + 1:40 v/v Gadoteric acid 0.5 mmol/mL (Dotarem^®^) at 4°C for at least three weeks to increase contrast. Then they were transferred to a solution of 1x PBS, 1:40 v/v Dotarem^®^ and 0.01% sodium azide and after two days a MRI scan was acquired.

### Magnetic resonance imaging

Images of the brain were acquired on a 7 Tesla PharmaScan^®^ (Bruker BioSpin, Ettlingen, Germany) equipped with a 370 mT/m gradient system with ParaVision^®^ 5.1 software. The skull was placed in a 15 mL Falcon™ tube filled with a proton-free fluid, Fomblin^®^ (Solvay, Belgium), to prevent susceptibility artefacts induced by tissue-air interfaces. A 23-mm diameter volume coil was used to acquire high resolution 3-dimentional gradient echo (FLASH) images with echo time 5.3 ms; repetition time = 15 ms; flip angle = 30°; FOV = [18 x 13 x 13]mm; zero-filling = 1.34; image matrix size = [256 x 186 x 186]; acquisition matrix size = [256 x 140 x 140]; isotropic spatial resolution = 0.070 mm; number of averages = 12; number of dummy scans = 10; receiver bandwidth = 50,000 Hz.

Four mice were excluded from analysis; two *mdx* and two *mdx-Xist*^Δhs^ mice. The brain of one *mdx* mouse was not completely located inside the field of view and the scans of the other three mice contained imaging artefacts. All remaining brains were successfully processed.

### Image registration and brain volumetric analysis

The registration scheme included registration of each subject brain to a template brain. For the template brain, volumes of interest (VOIs) for the whole brain and 22 anatomical structures were manually segmented based on the Waxholm mouse brain atlas [44], the Franklin and Paxinos atlas [45,46] and the Allen Brain Atlas [47] using AMIRA (v5, FEI Software, Oregon, USA) [48]. Next to this, the 22 anatomical structures were either classified as white or grey matter, according to S1 Table. The whole brain volume was defined as the brain tissue limited caudally by the cerebellum and rostrally by the rhinal fissure. Using the information provided by the inverse deformation field for each subject-to-template registration, the template VOIs were propagated to the individual datasets, enabling quantitative comparison of corresponding areas. The VOIs were evaluated quantitatively for volume change.

Two independent observers verified the quality of the registration by visual inspection, using a custom-made graphic user interface built with MeVisLab (v2.7, MeVis Medical Solutions AG, Bremen, Germany) [49]. The registration was implemented using the open source image registration toolbox Elastix [50] and performed in a coarse-to-fine process. Initially, rigid registration was performed to compensate for translation and rotation. Afterwards, an affine registration was conducted to compensate for differences in brain size, followed by a non-rigid B-spline registration to compensate for local changes. A Gaussian image pyramid was employed in all registration steps, applying four resolutions for the rigid and B-spline and two for the affine registration. Mutual information was used as a similarity metric. The two independent observers were not blinded; however, no manual alterations were needed as the registration of all subject brains passed the quality control. Detailed information on the used registration parameters can be found on the Elastix website (http://elastix.bigr.nl/wiki/index.php/Par0033).

### Skull morphology analysis

To assess skull morphology, the ratios of the length of the major and minor axis of the skull of *mdx* and BL10 mice were assessed on axial and coronal MRI planes in Osirix Lite v7.5. This ratio is directly related to skull eccentricity which is known to be different in DMD patients. The coronal plane was set to mid-corpus callosum and the axial plane was rostrally set to the mid-olfactory bulb and caudally set to the second white matter branching of the arbor vitae in the cerebellum (S1 Fig).

### Dystrophin quantification

Dystrophin levels were determined by means of Western blot. In total seven *mdx-Xist*^Δhs^ mice brains were measured. The brain tissue was homogenised in 400 μl protein isolation buffer (20% SDS and 100 mM Tris-HCl pH 6.8) in the MagNA Lyser (Roche Diagnostics, The Netherlands). After centrifuging (4°C, 20.817 rcf, 10 minutes), the supernatant was removed and the pellet resuspended in 200 μl protein isolation buffer. An aliquot was used to determine protein concentration with the BCA protein assay kit (Thermo Scientific, USA).

Sample buffer (75 mM Tris-HCl pH 6.8, 15% SDS, 20% glycerol, 5% β-mercaptoethanol, 0.0008% bromophenol blue) was added to the samples and the size marker (HiMark Pre-stained Protein Standard, Thermo Scientific, USA), which were then boiled at 90°C for five minutes. Total protein (60 μg) was loaded onto a 3–8% Criterion XT Tris-Acetate gel (Bio-Rad, USA) and a calibration curve was made by diluting wild type lysate in *mdx* protein lysate. The gel was run at 75V for one hour and at 150V for one and a half hour consecutively, the buffer was refreshed at the voltage change. The gel was then blotted onto a nitrocellulose membrane with the Trans-Blot Turbo system (Bio-Rad, USA) at 2.5A for 10 minutes. The blot was incubated in blocking buffer (5% non-fat dried milk (Elk, Campina, The Netherlands) in Tris Buffered Saline (TBS) with 0.005% Tween20 (TBST)) for one hour. Afterwards the blot was washed (15 minutes in TBST) and incubated overnight with the primary antibody NCL-DYS1 (dilution 1:50, Novocastra, UK) to detect dystrophin and with anti-Post Synaptic Density Protein 95 (dilution 1:5000, Millipore, USA) as a loading control in TBS. The blot was washed in TBST and incubated with the secondary antibody IRDye 800CW goat-anti-mouse IgG (dilution 1:5000, Li-Cor, USA) for one hour. Blots were washed (two times for 20 minutes in TBST and one time for 20 minutes in TBS) and visualized with the Odyssey system (Li-Cor, USA).

### RT-qPCR analysis

Total RNA was isolated from the whole brain with TRIsure (LifeGene, Israel) and purified with the NucleoSpin RNA II kit including a DNAse digestion (Bioke, the Netherlands) according to the manufacturer’s instructions. RNA concentration was measured on a Nanodrop (Nanodrop Technologies, USA). cDNA was synthesised with random hexamer primers and gene expression levels were determined by Sybr Green based Real Time qPCR (95°C 10 sec, 60°C 30 sec, 72°C 20 sec. 50 cycles followed by melting curve determination) on the Roche Lightcycler 480 (Roche diagnostics Ltd, UK). Expression of *Dp427c*, *Dp427p* and *Dp71* were analysed, together with the *Rpl22* housekeeping gene which was used as reference. Primer efficiencies were determined with LinREgPCR version 11.1 [51]. The Cp values were obtained with the second derivative maximum method and analysed. The used primer sequences are given in S2 Table.

### Statistics

A one-way ANOVA analysis with a Tukey’s post-hoc test was performed to compare body mass, whole brain volume and gene expression among all strains. Genetic background appeared to have a large impact on whole brain volume, preventing direct comparisons between all strains. Therefore, *mdx* mice were directly compared to BL10 mice (both BL10 background) and *mdx-Xist*^Δhs^ to BL10*-Xist*^Δhs^ mice (both a mixed BL10 and BL6 background).

The 22 brain structures, grey and white matter were compared using Welch’s T-tests. To correct the 22 brain structures for multiple comparisons, the *P-*values were corrected using the false discovery rate method by Benjamini and Hochberg [52,53]. The false discovery rate was set to 5%, defined as the expected maximum rate of the tested structures to be false positive. R (3.3.0) [54] in RStudio (1.0.143) [55] was used for all statistical tests.

## Results

**Fig 1:**
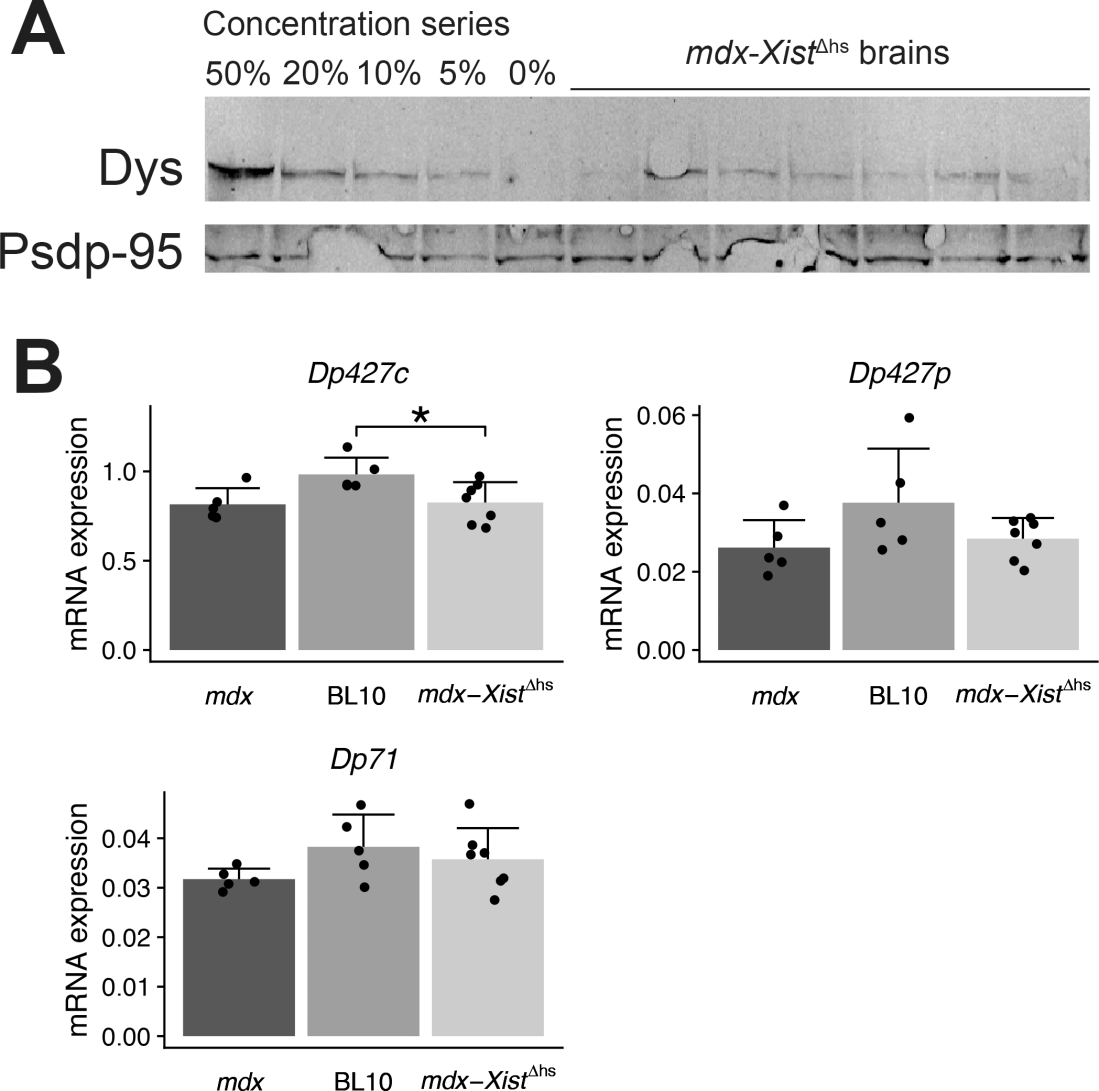
Dystrophin expression in *mdx-Xist*^Δhs^ mice. **A:** Representative Western blot of *mdx-Xist*^Δhs^ brains revealed that mice expressed low levels of full-length dystrophin in their brain. Levels varied between individuals but were below 20% in all mice. Post Synaptic Density Protein 95 (Psdp95) served as a loading control. Wildtype samples for the concentration series were diluted in *mdx* protein lysate to ensure equal protein loading of all samples. **B:** mRNA expression levels of neither full-length nor Dp71 differed between *mdx* and BL10 mice. However, expression of Dp427c was higher in BL10 compared to *mdx*-*Xist*^Δhs^ mice. Error bars represents the standard deviation. Asterisk indicates *P*<0.05.

To study the effects of dystrophinopathy on brain morphology, we acquired post-mortem MRI scans of fixated brains of two different DMD mouse models (*mdx* and *mdx-Xist*^Δhs^ mice) and genetically corresponding wild type strains. The fixation procedure of the brains impeded us to assess full-length dystrophin levels in the *mdx-Xist*^Δhs^ tissues. We therefore confirmed in another cohort of *mdx-Xist*^Δhs^ mice low full-length dystrophin protein levels in the brain (Fig 1A). In addition, we confirmed full-length (Dp427c and p) and Dp71 mRNA expression in all strains. Dp427c expression was higher in BL10 compared to *mdx*-*Xist*^Δhs^ mice (Fig 1B), but not compared to *mdx* mice (*P* = 0.052), while the expression of other isoforms was comparable between strains.

The MRI scans revealed that volumes of the whole brain and of the 22 individual brain structures were comparable between the groups (Table 1). The whole brain volume of *mdx* mice was not significantly different compared to BL10 mice (464 ± 14.3 mm^3^ *vs* 457 ± 9.8 mm^3^). Volumes of the 22 segmented structures were normalized to whole brain volume instead of body mass, as the latter was not equally distributed (*P* = 0.02) among the four strains. *Mdx* mice had a higher body mass compared to both wild type and *mdx-Xist*^Δhs^ mice (Table 1). This difference is associated with the pathological muscle hypertrophy in *mdx* mice at the age of 12 weeks [56] and not due to growth abnormalities.

**Table 1:** Volumes (in mm^3^) of 22 brain structures in mouse models of Duchenne muscular dystrophy. The volumes of 22 segmented brain structures in *mdx*, BL10, *mdx*-*Xist*^Δhs^ and BL10-*Xist*^Δhs^ mice are presented in mm^3^. Body mass was measured just before sacrificing the mice, except for two *mdx-Xist*^Δhs^ mice of which body mass was not obtained. The volumes of these 22 structures were normalized to whole brain volume and compared using Welch’s T-tests within each genetic background (*mdx vs* BL10 and *mdx*-*Xist*^Δhs^ *vs* BL10-*Xist*^Δhs^) and corrected for multiple comparisons using the false discovery rate. The corresponding *P*-values are presented and structures which are significantly different in volume are written in bold. The hippocampus, globus pallidus, caudate putamen and hypothalamus were different between *mdx* and BL10 mice. Thirteen structures were different between *mdx*-*Xist*^Δhs^ and BL10-*Xist*^Δhs^ mice, consisting of a mixture of both grey and white matter structures.

	*Mdx*			BL10			*P*-value	*mdx*-*Xist*^Δhs^			BL10-*Xist*^Δhs^			*P*-value
Number of included mice	8			7				7			9			
Body mass (g)	26.58	±	2.15	23.47	±	1.08	**0.004**	23.10	±	0.73	24.60	±	2.74	0.153
Whole brain	464.067	±	14.289	457.096	±	9.762	0.286	501.533	±	13.350	498.019	±	18.551	0.666
Grey matter	267.935	±	7.113	259.806	±	4.427	**0.027**	289.803	±	8.517	279.584	±	9.808	**0.000**
White matter	20.232	±	0.749	19.270	±	0.680	**0.059**	22.194	±	0.943	19.653	±	1.031	**0.000**
Hippocampus	**25.351**	**±**	**1.293**	**23.581**	**±**	**0.550**	**0.018**	**27.218**	**±**	**1.254**	**25.965**	**±**	**1.047**	**0.046**
Globus pallidus	**1.676**	**±**	**0.086**	**1.507**	**±**	**0.051**	**0.027**	**2.011**	**±**	**0.056**	**1.747**	**±**	**0.069**	**0.000**
Caudate putamen	**23.509**	**±**	**0.804**	**21.904**	**±**	**0.433**	**0.026**	**26.417**	**±**	**0.892**	**23.398**	**±**	**0.475**	**0.000**
Hypothalamus	**8.576**	**±**	**0.253**	**8.801**	**±**	**0.330**	**0.044**	9.183	±	0.391	9.139	±	0.563	0.905
Anterior commissure	0.921	±	0.025	0.856	±	0.030	0.083	**1.006**	**±**	**0.038**	**0.903**	**±**	**0.044**	**0.001**
Periaqueductal gray	3.152	±	0.146	2.976	±	0.138	0.113	**3.686**	**±**	**0.236**	**3.309**	**±**	**0.207**	**0.009**
Internal capsule	1.562	±	0.100	1.443	±	0.063	0.101	**1.885**	**±**	**0.087**	**1.568**	**±**	**0.079**	**0.000**
Fornix	0.200	±	0.006	0.187	±	0.012	0.188	0.220	±	0.014	0.205	±	0.012	0.098
Amygdala	8.716	±	0.418	8.301	±	0.248	0.194	**9.602**	**±**	**0.368**	**9.019**	**±**	**0.494**	**0.002**
Corpus callosum	13.933	±	0.578	13.287	±	0.434	0.196	**15.181**	**±**	**0.647**	**13.640**	**±**	**0.754**	**0.000**
Septal nucleus	4.194	±	0.113	3.946	±	0.258	0.182	**4.444**	**±**	**0.297**	**3.835**	**±**	**0.203**	**0.002**
Olfactory bulb	26.803	±	1.710	27.402	±	0.758	0.197	28.378	±	0.843	28.970	±	2.165	0.330
Interpeduncular nucleus	0.223	±	0.015	0.228	±	0.010	0.248	0.239	±	0.006	0.228	±	0.020	0.302
Nucleus accumbens	3.799	±	0.045	3.663	±	0.118	0.311	**4.060**	**±**	**0.171**	**3.772**	**±**	**0.175**	**0.002**
Fimbria	3.617	±	0.188	3.497	±	0.248	0.764	**3.903**	**±**	**0.281**	**3.336**	**±**	**0.231**	**0.002**
Inferior colliculus	5.218	±	0.077	5.075	±	0.323	0.720	6.220	±	0.215	6.081	±	0.338	0.456
Ventricles	1.191	±	0.025	1.162	±	0.055	0.687	**1.291**	**±**	**0.083**	**1.178**	**±**	**0.074**	**0.021**
Cortex	143.851	±	3.298	140.930	±	2.608	0.653	151.010	±	4.942	149.281	±	6.529	0.623
Midbrain thalamus	38.406	±	1.290	37.941	±	0.910	0.726	**43.735**	**±**	**1.465**	**42.048**	**±**	**1.215**	**0.004**
Superior colliculus	8.457	±	0.413	8.393	±	0.336	0.700	9.614	±	0.375	9.408	±	0.521	0.413
Cerebellum	51.859	±	2.532	50.917	±	2.050	0.833	58.717	±	2.073	59.774	±	3.028	0.116
Substantia nigra	1.383	±	0.067	1.362	±	0.044	0.992	1.547	±	0.070	1.493	±	0.045	0.189

**Fig 2:**
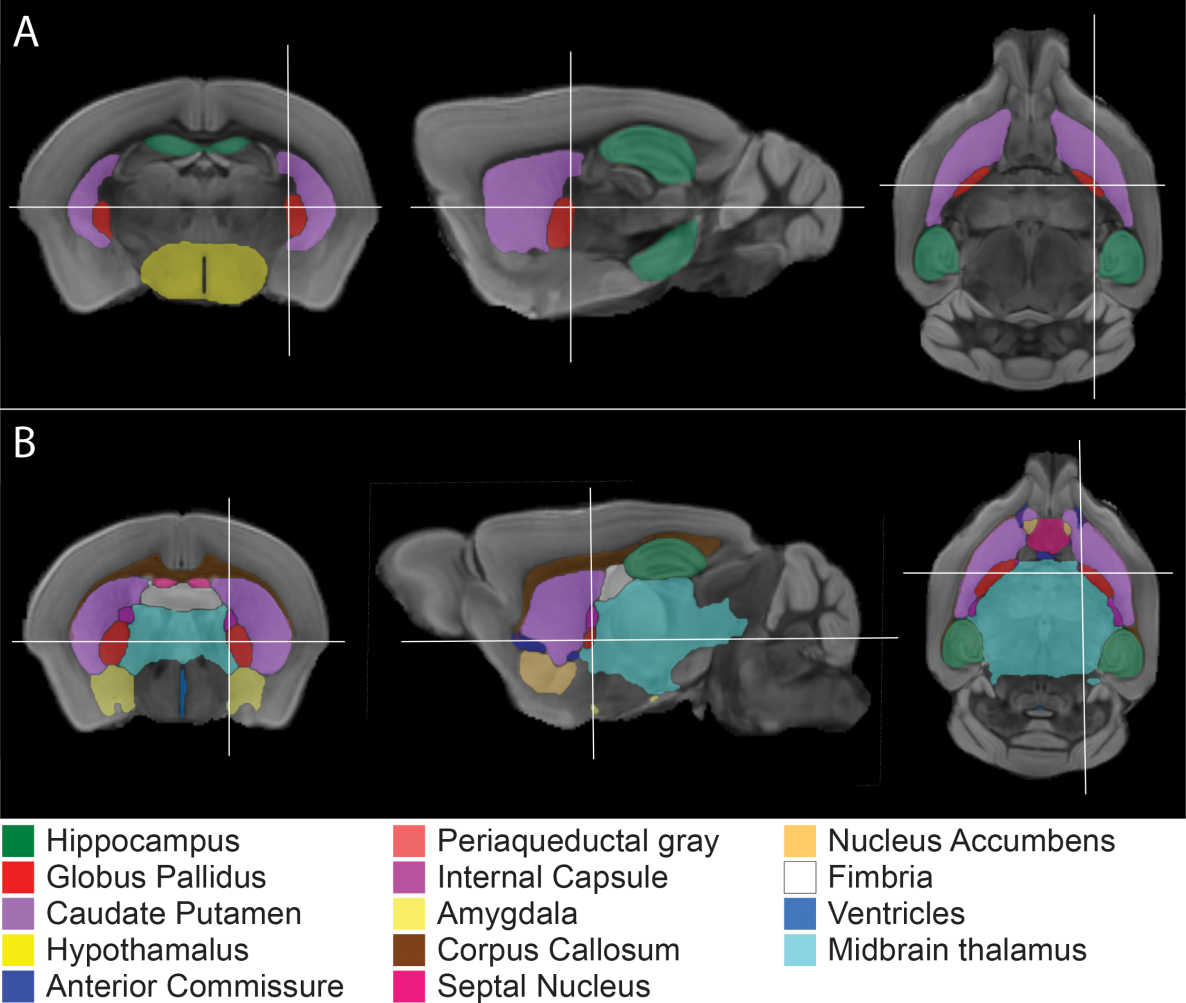
The volumes of 22 segmented brain structures in *mdx*, BL10, *mdx*-*Xist*^Δhs^ and BL10-*Xist*^Δhs^ mice were compared within each genetic background (*mdx vs* BL10 and *mdx*-*Xist*^Δhs^ *vs* BL10-*Xist*^Δhs^). The brain structures found significantly different in volume after false discovery rate correction are shown and consisted of both grey and white matter structures for both comparisons. Coronal, sagittal and axial planes are shown indicated by white lines. **A:** Comparison between *mdx* and BL10 showed four different brain structures: the hippocampus, globus pallidus, caudate putamen and hypothalamus. **B:** Comparison between *mdx*-*Xist*^Δhs^ and BL10-*Xist*^Δhs^ showed 13 different structures.

Four out of the 22 brain structures were significantly different in volume in *mdx* mice compared to BL10 controls after adjustment with the false discovery rate, as shown in Table 1. These structures consisted of both grey and white matter: the hippocampus, globus pallidus and caudate putamen were larger, while the hypothalamus was smaller (Fig 2A). When grouped to grey and white matter, only grey matter was significantly larger in *mdx* mice.

In mice with a genetic mixed BL10/BL6 background (*mdx-Xist*^Δhs^ and BL10-*Xist*^Δhs^), significantly larger whole brain volumes were found compared to mice with a pure BL10 background (*mdx* and BL10). In *mdx-Xist*^Δhs^ mice, 13 of the 22 brain structures were significantly increased in volume compared to BL10-*Xist*^Δhs^ mice (Fig 2B) after false discovery rate adjustments. The same brain structures enlarged in *mdx* mice were also enlarged in *mdx*-*Xist*^Δhs^ mice, except for the hypothalamus.

We compared the above MRI findings to known full-length dystrophin expressing regions in mice: the cortex, hippocampus, cerebellum and amygdala [31]. Out of all these dystrophin-expressing structures only the hippocampus was significantly larger in *mdx* (*P* = 0.018) and *mdx*-*Xist*^Δhs^ (*P* = 0.046) mice, compared to their wild type counterparts. The other full-length dystrophin-expressing structures (cortex and cerebellum) did not show differences in volume (cortex *P* = 0.653 and *P* = 0.623, cerebellum *P* = 0.833 and *P* = 0.116, respectively for *mdx* and *mdx*-*Xist*^Δhs^ mice), while the amygdala was only significantly larger in *mdx*-*Xist*^Δhs^ mice *(P* = 0.002).

Lastly, we analyzed skull morphology in *mdx* and BL10 mice to assess skull eccentricity, which is altered in DMD patients [30]. No differences were found in skull eccentricity between *mdx* and wild type mice (*P* = 0.50 for the axial and *P* = 0.33 for the coronal plane (S1 Fig)).

## Discussion

This study utilized high resolution MRI to investigate volumes of individual brain structures in DMD mouse models in detail. Whole brain volume was not significantly increased in *mdx* mice compared to BL10 mice. This is in line with two previous MRI studies, where six-month-old *mdx* and BL10 mice had a whole brain volume of 473.19 ± 9.23 mm^3^ and 465.87 ± 6.82 mm^3^ respectively [40]. A study in 12-month-old *mdx* and BL10 mice found a non-significant increase in whole brain volume normalized to body mass [41]. While both previous studies and this study found a mild increase in brain volume, none of these differences were significant, which may be attributed to the small sample size (n = 8, n = 15 and n = 17 respectively).

The low amounts of full-length dystrophin protein in the whole brain of *mdx*-*Xist*^Δhs^ mice were in agreement with a previous study which quantified dystrophin protein in heart tissue [57], also an organ with minimal cell renewal, showing varying dystrophin levels between 3 and 15%. Messenger RNA levels of both full-length and Dp71 dystrophin were not significantly different between *mdx* and BL10 mice, though it tended to be lower in *mdx* mice, which was also found by Perronnet et al. [58]. We contribute the detected brain volume differences in this study to the absence or low amounts of full-length dystrophin, since no clear difference was observed for Dp71 expression levels.

In contrast to previous studies [40,41], we segmented the volumes of 22 brain structures. We observed wide spread structural changes, consisting of both grey and white matter structures, in mice lacking dystrophin and in mice with low levels of dystrophin compared to their genetic background matched controls. From the full-length dystrophin expressing regions in mice, volumes of the cortex, cerebellum and amygdala were comparable to wild type values, while the hippocampus was increased in size. This implies that there is no direct relation between the lack of full-length dystrophin in these structures and possible macro-structural alterations.

To assess the consequences of the expression of low amounts of full-length dystrophin on brain morphology we investigated *mdx*-*Xist*^Δhs^ and the corresponding wild type BL10-*Xist*^Δhs^ mice. The mixed BL6 and BL10 background of these mice resulted in an 8.4% higher whole brain volume compared to *mdx* and BL10 mice with a pure BL10 background. These unexpected genetic background effects prevented direct comparisons of *mdx*-*Xist*^Δhs^ to *mdx* and BL10 mice.

Whole brain volume of *mdx*-*Xist*^Δhs^ mice was not increased as much as that of *mdx* mice, compared to their wild types (0.70% and 1.50% respectively). This implies that a small amount of full-length dystrophin might modulate whole brain volume towards wild type values. However, we still found wide-spread volumetric differences in the hippocampus and 12 other structures.

Other studies have provided evidence for wide-spread structural changes in *mdx* mice using diffusion weighted (DWI) and diffusion tensor (DTI) MRI studies [59,60], although the findings are conflicting. DWI/DTI captures microscopic changes by estimating the amount of water diffusion, restricted by e.g. cell membranes, by estimating the apparent diffusion coefficient (ADC). Goodnough *et al*. found significantly lower ADC values in whole brain (without ventricles) in 2-month-old and 10-month-old *mdx* mice compared to C57BL/6 mice [61]. They hypothesized that the found ADC reduction was caused by cellular edema, caused by impaired brain vasculature. The mildly increased brain volume of *mdx* mice found in our study could be the result of edema as well. Xu *et al*. used *ex vivo* DTI localized to the prefrontal cortex, hippocampus and cerebellum in 9-month-old *mdx* mice. In *mdx* mice a significant increase in ADC values in the prefrontal cortex and decrease of fractional anisotropy in the hippocampus was found. No differences in diffusion parameters were found in the cerebellum. This is consistent with our data as we did not find any volume changes in the cerebellum, while the hippocampus was enlarged in *mdx* and *mdx*-*Xist*^Δhs^ mice.

In contrast with the trend of increased brain volume in *mdx* mice, mild cerebral atrophy has been found in DMD patients [62,63]. A recent MRI study by Doorenweerd *et al*. showed non-significant (*P* = 0.056) reduction in whole brain volume between patients and controls [29]. However, their study divided patients into two groups; patients lacking Dp427 (Dp140^+^) and patients lacking both Dp427 and Dp140 (Dp140^-^). Only in Dp140^-^ patients a significant reduction in whole brain volume was found compared to controls. Based on these findings it would be interesting to acquire volume data from *mdx*^*4cv*^ mice, lacking both Dp140 and Dp427, to investigate the effect of Dp140 on brain volumes in mice. An alternative explanation of the reduced brain volumes in DMD patients could however be the influence of steroid use. Brain atrophy has also been shown in imaging studies of multiple sclerosis and asthma patients treated with corticosteroids or patients with Cushing disease, which is characterized by excess of endogenous steroid production [64–69]. As 24 out of 29 DMD patients in the study were on steroid treatment, Doorenweerd *et al*. could not rule out this confounding effect. Next to this, recent work [15] showed an devoid of full-length dystrophin (Dp427p) expression in the human cerebellum, however mice do express Dp427p in their cerebellum, so it might be that dystrophin plays a different role in mice.

In DMD patients, the differences in skull shape, observed as reduced skull eccentricity, are a robust and prominent finding [30]. However, the skull morphology of *mdx* mice was not found to be different from controls. A possible explanation is that the skull shape is primarily determined by the craniofacial muscles and their deterioration due to DMD. Both in humans and in mice, a clear association exists between craniofacial muscle strength, measured as bite force, and craniofacial morphology [70,71]. In DMD patients bite force is reduced [72,73], indicating reduced craniofacial muscle strength, while in *mdx* mice bite force is not affected [74].

A limitation of our study is the lack of temporal information, as only 3-month-old mice were investigated. It is likely that the absence of full-length dystrophin results in aberrations during developmental processes, leading to wide-spread structural changes. Therefore, for future studies it would be interesting to obtain measurements during the process of maturation, to elucidate how the absence of full-length dystrophin influences brain development. Secondly, additional mouse models lacking different and multiple dystrophin isoforms should be used to elucidate the complex effects of the various isoforms on brain structure, function and development.

## Supporting information

S1 Fig Skull morphology was assessed on axial and coronal planes. **No differences between *mdx* and BL10 mice were found, while DMD patients present with aberrant skull morphology.** To assess skull morphology, the ratios of the length of the major and minor axis of the skull of *mdx* and BL10 mice were assessed on axial (C) and coronal (B) MRI planes in Osirix Lite v7.5. This ratio is directly related to skull eccentricity which is known to be different in DMD patients. The coronal plane was set to mid-corpus callosum (A) and the axial plane was rostrally set to the mid-olfactory bulb and caudally set to the second white matter branching of the arbor vitae in the cerebellum (A). No significant differences were found between *mdx* (D+E) and BL10 mice (F+G).
(TIF)

S1 Table Grey and white matter structures. The 22 segmented anatomical structures were either classified as white or grey matter.
(XLSX)

S2 Table qPCR primer sequences. (XLSX)
