# Supplementary figures and images for: Influence of full-length dystrophin on brain volumes in mouse models of Duchenne muscular dystrophy
Source: PLoS One. 2018 Mar 30;13(3):e0194636. doi: 10.1371/journal.pone.0194636 (PMC5877835; doi:10.1371/journal.pone.0194636)

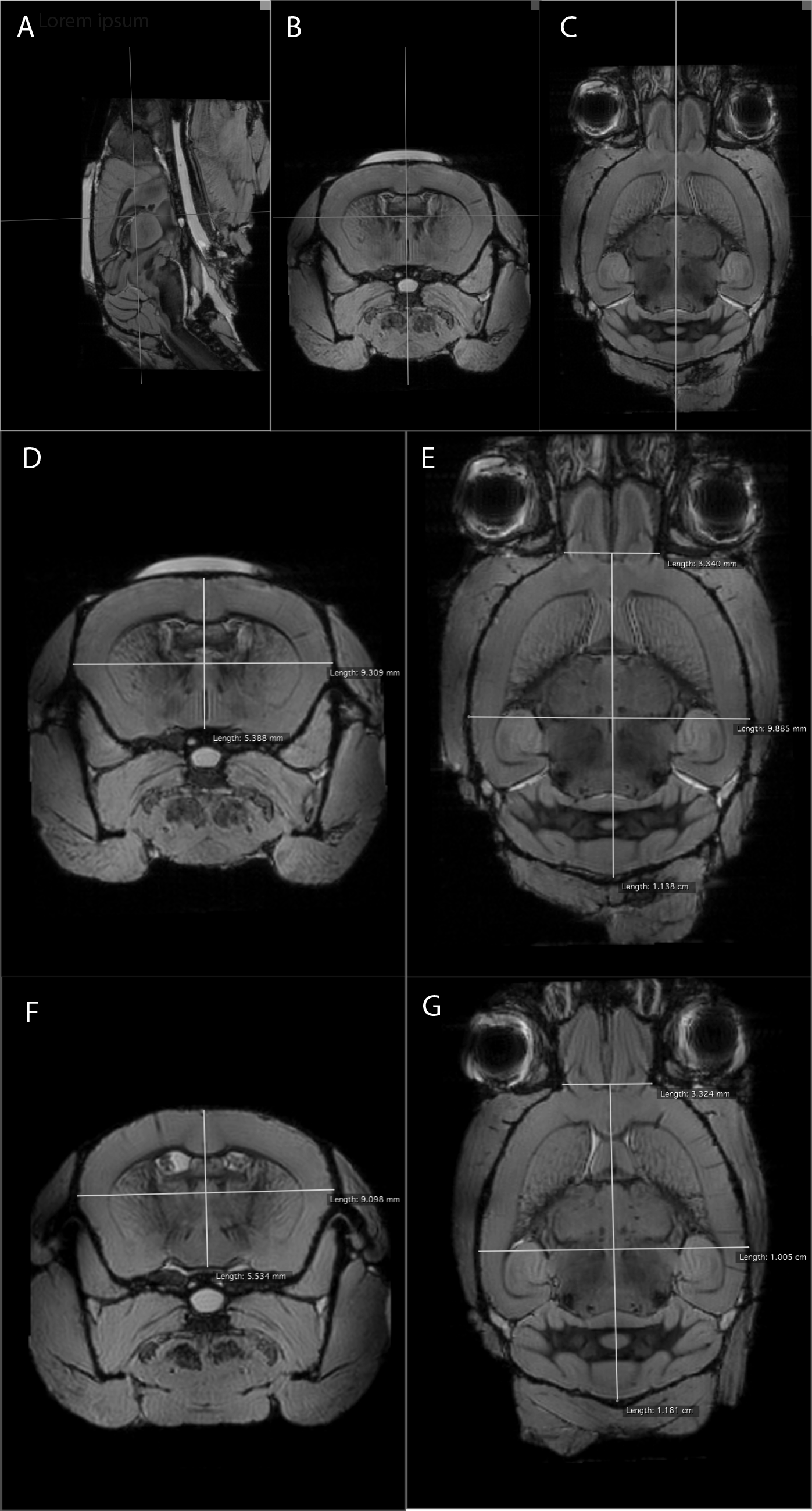

Supplement: S1 Fig — No differences between mdx and BL10 mice were found, while DMD patients present with aberrant skull morphology. To assess skull morphology, the ratios of the length of the major and minor axis of the skull of mdx and BL10 mice were assessed on axial (C) and coronal (B) MRI planes in Osirix Lite v7.5. This ratio is directly related to skull eccentricity which is known to be different in DMD patients. The coronal plane was set to mid-corpus callosum (A) and the axial plane was rostrally set to the mid-olfactory bulb and caudally set to the second white matter branching of the arbor vitae in the cerebellum (A). No significant differences were found between mdx (D+E) and BL10 mice (F+G). (TIF) [file pone.0194636.s001.tif]
